# Immunomodulatory effects of soluble CD5 on experimental tumor models

**DOI:** 10.18632/oncotarget.22564

**Published:** 2017-11-20

**Authors:** Inês T. Simões, Fernando Aranda, Esther Carreras, Maria Velasco-de Andrés, Sergi Casadó-Llombart, Vanesa G. Martinez, Francisco Lozano

**Affiliations:** ^1^ Immunoreceptors of the Innate and Adaptive System, Institut d’Investigacions Biomèdiques August Pi i Sunyer, 08036, Barcelona, Spain; ^2^ Servei d’Immunologia, Centre de Diagnòstic Biomèdic, Hospital Clínic de Barcelona, 08036, Barcelona, Spain; ^3^ Departament de Biomedicina, Facultat de Medicina, Universitat de Barcelona, 08036, Barcelona, Spain

**Keywords:** CD5, immunotherapy, immune-checkpoint, T regulatory cells, NK cells

## Abstract

Modulation of antitumor immune responses by targeting immune checkpoint regulators has been proven successful in the treatment of many different tumors. Recent evidence shows that the lymphocyte receptor CD5 –a negative regulator of TCR-mediated signaling- may play a role in the anti-tumor immune response. To explore such an issue, we developed transgenic C57BL/6 mice expressing a soluble form of human CD5 (shCD5EμTg), putatively blocking CD5-mediated interactions (“decoy receptor” effect). Homozygous shCD5EμTg mice showed reduced growth rates of tumor cells of melanoma (B16-F0) and thymoma (EG7-OVA) origin. Concomitantly, increased CD4^+^ and CD8^+^ T cell numbers, as well as reduced proportion of CD4^+^CD25^+^FoxP3^+^ (T_reg_) cells were observed in tumor draining lymph nodes (TdLN). TdLN cell suspensions from tumor-bearing shCD5EμTg mice showed increased both tumor specific and non-specific cytolitic activity. Moreover, subcutaneous peritumoral (*p.t.*) injection of recombinant shCD5 to wild-type (WT) mice slowed B16-F0 tumor growth, and reproduced the above mentioned TdLN cellular changes. Interestingly, lower intratumoral IL-6 levels –an inhibitor of Natural Killer (NK) cell cytotoxity- were observed in both transgenic and rshCD5-treated WT mice and the anti-tumor effect was abrogated by mAb-induced NK cell depletion. Taken together, the results further illustrate the putative regulatory role of CD5-mediated interactions in anti-tumor immune responses, which would be at least in part fostered by NK cells.

## INTRODUCTION

Cancer immunotherapy has taken advantage of either potentiating or inhibiting several immune cell surface receptors that modulate the intensity of the immune response –the so-called immune checkpoint regulators. Impairing the function of immune checkpoint inhibitors enhances ongoing immune responses and helps the host immune system to more efficiently eradicate exogenous (e.g., infection) or endogenous (e.g., cancer) challenges.

CD5 is a lymphoid member of the scavenger receptor cysteine-rich (SRCR) superfamily [1], which is expressed by all T cells and a small subset of mature B cells (B1a) [2]. CD5 physically associates with the antigen-specific receptor complex present on T [3, 4] and B [5] cells (TCR and BCR, respectively) and co-localizes with it at the center of the T-Antigen presenting cell (APC) immune synapse [6, 7]. While initially considered a co-stimulatory receptor, studies in *knock-out* mice [8, 9] unequivocally demonstrated the negative modulatory role of CD5 on TCR/BCR signaling. Accordingly, thymocytes and B1a cells from CD5-deficient mice are hyper-responsive to TCR and BCR cross-linking, resulting in higher proliferation, mobilization of intracellular calcium and phosphorylation of several signaling proteins [8, 9]. Interestingly, T and B cell populations with regulatory functions (T_reg_ and B10 cells, respectively) express high surface CD5 levels, which in turn might be important to their generation and/or function [10, 11]. Indeed, CD5-deficient mice display higher number of natural T_reg_ cells, although their suppressive activity is a matter of controversy [12, 13].

A number of studies have reported the putative involvement of CD5 in the modulation of antitumor responses, this including the effective treatment with a non-depleting anti-CD5 (Lyt-1) monoclonal antibody (mAb) [14], and the adaptation of human CD5 expression levels in tumor infiltrating lymphocytes (TILs) [15, 16]. Moreover, CD5-deficient mice engrafted with B16-F10 melanoma cells displayed slower tumor growth compared to wild-type (WT) C57BL/6 mice, which was associated with tumor infiltration by T cells exhibiting a more activated phenotype and enhanced antitumor effector functions [17]. Latest evidence also comes from human genetic studies showing that functionally relevant CD5 variants are of prognostic value in cancer patients undergoing melanoma or chronic lymphocytic leukemia [18, 19].

The ultimate nature of the CD5 ligand/s is still a controversial issue due to the lack of intergroup reproducibility of the proposed ones (CD72, framework region of IgV_H_, gp200, gp40-80, gp150, CD5 itself or IL-6) [20, 21] imposing some constraints in deciphering the ultimate physiological function/s of CD5. In an attempt to unveil the role played by the receptor-ligand interaction/s mediated by CD5 in lymphocyte physiology, our group took advantage of the existence of a soluble form of human CD5 (shCD5) and the interspecies conservation of the CD5-ligand interaction/s. As reported for other lymphocyte surface receptors [22], shCD5 is detected at low concentrations (pg/mL range) in the serum of normal individuals, resulting from proteolytic cleavage upon lymphocyte activation [23]. Accordingly, a transgenic mouse line constitutively expressing higher sustained serum levels (ng/mL range) of shCD5 (shCD5EμTg) was developed [24]. This circulating shCD5 form was expected to interact with the CD5 ligand/s, thus interfering with the receptor-ligand interactions (decoy receptor effect), and resulting in a functional knockdown of CD5. Preliminary analysis of heterozygous shCD5EμTg mice showed a decreased frequency of spleen T_reg_ and peritoneal IL-10 producing B-cell (B10) populations together with an increased proportion of spleen Natural Killer T cells (NKT) [24]. Concomitantly, such mice showed exacerbated forms of different experimental mouse models of autoimmune diseases (Collagen-Induced Arthritis and Experimental Autoimmune Encephalitis) as well as enhanced anti-tumor immune responses against B16 melanoma cells [24], thus supporting the importance of CD5 in the regulation of peripheral immune responses.

To further assess the potential of the CD5 lymphocyte receptor as a putative target of immunomodulation in cancer, we have pursued the analysis of the local anti-tumor response in homozygous shCD5EμTg mice. These mice showed significantly slower tumor growth when challenged with isogenic tumor cell lines of different lineages (melanoma and lymphoma). The analysis of tumor draining lymph nodes (TdLNs) showed lymphocyte subset changes compatible with increased anti-tumor immune responses. Importantly, most of the observations made with shCD5EμTg mice were reproduced by subcutaneous peritumoral (*p.t.*) injection of recombinant shCD5 (rshCD5) to tumor-challenged WT mice, thus excluding the possibility of transgenesis artifacts and strongly supporting the immunomodulatory properties of shCD5 in cancer.

## RESULTS

### shCD5EμTg mice display enhanced anti-tumoral responses to certain tumor types

**Figure 1:**
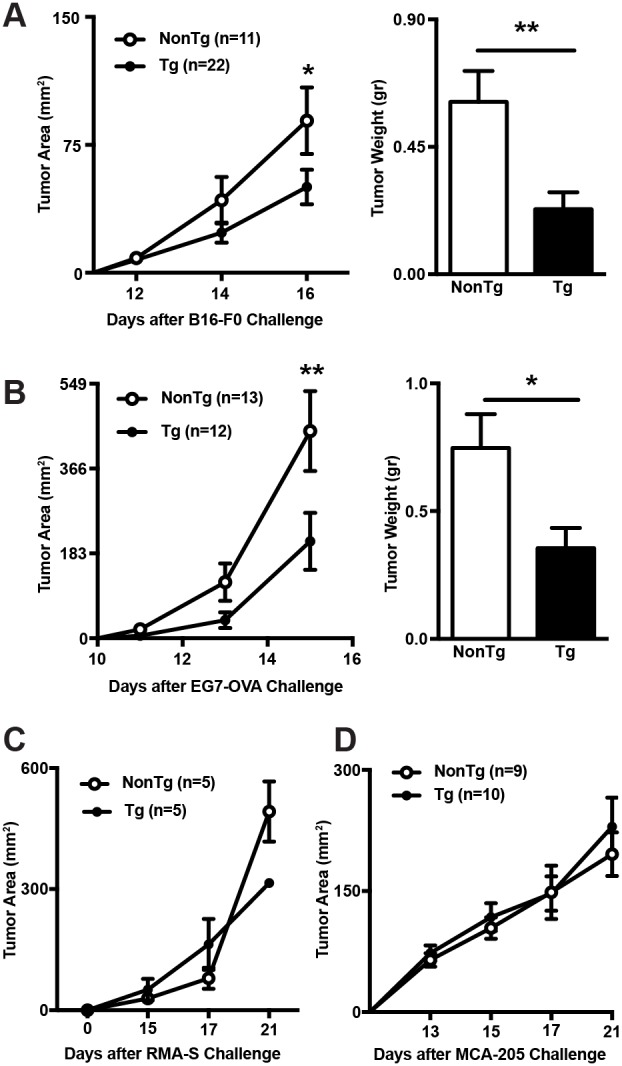
Comparison of tumor growth rates between shCD5EμTg and NonTg mice **(A)** shCD5EμTg (n = 22) and NonTg mice (n = 11) were injected *s.c.* with 5×10^4^ B16-F0 melanoma cells and tumor area measured every other day **(left panel)**. Tumor weight at day 16 is also represented **(right panel)**. **(B)** Same as in A but injecting *s.c.* shCD5EμTg (n = 12) and NonTg (n = 13) mice with 5×10^4^ EG7-OVA cells. **(C)** Tumor area curve from shCD5EμTg mice (n = 5) and NonTg (n = 5) injected *s.c.* with 5×10^4^ RMA-S cells. **(D)** Tumor area curve from shCD5EμTg (n = 10) and NonTg (n = 9) mice injected *s.c.* with 5×10^4^ MCA-205 cells. Values are represented as mean ± SEM. ^*^p< 0.05; ^**^p< 0.01 (unpaired *t* test).

In accordance with previous results from heterozygous shCD5EμTg mice [24], the homozygous mice kept under conventional housing conditions showed statistically significant reduced tumor growth rates and tumor weight when challenged with isogenic B16-F0 melanoma cells, as compared with non-transgenic (NonTg) controls (Figure 1A). Similar results were observed when the anti-tumor response of shCD5EμTg mice was evaluated against EG7 lymphoma cells stably expressing ovalbumin (EG7-OVA) (Figure 1B). On the contrary, the tumor growth rates did not differ between shCD5EμTg and NonTg mice when injected with RMA-S lymphoma cells (a major histocompatibility complex class I-deficient RMA variant) (Figure 1C), MCA-205 sarcoma cells (Figure 1D), and MC-38 colon carcinoma cells (data not shown). These results indicate that shCD5EμTg mice exhibit an improved non-specific anti-tumor response, which is nevertheless restricted to certain tumor cell types.

### shCD5EμTg mice display increased lymphoid cell numbers in TdLN

**Figure 2:**
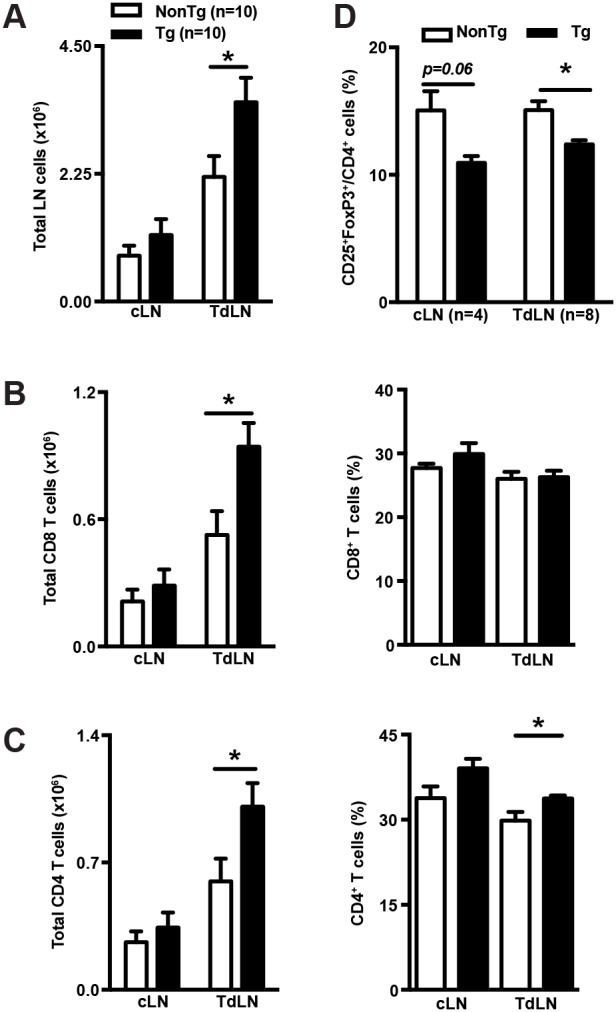
shCD5EμTg mice display increased lymphoid cell numbers in TdLN **(A)** Total cell numbers from cLN and TdLN of shCD5EμTg (n = 10) and NonTg (n = 10) mice challenged with 5×10^4^ B16-F0 cells were counted from single cell suspensions. **(B-C)** Total numbers and percentage of CD8^+^ (B) and CD4^+^ (C) T cells from cLN and TdLN from the same mice as in A. **(D)** Percentage of CD25^+^FoxP3^+^ cells in CD4^+^ from cLN and TdLN of shCD5EμTg (n = 8) and NonTg (n = 8) mice challenged with B16-F0 cells. Values are represented as mean ± SEM in a conventional mice facility. ^*^p< 0.05 (unpaired *t* test).

Further characterization of the anti-tumor response was performed by comparing the lymphocyte subset composition of tumor draining (TdLN) and contra-lateral (cLN) lymph nodes in B16-F0-challenged shCD5EμTg and NonTg mice. As shown by Figure 2A, TdLN but not cLN from shCD5EμTg mice, showed a statistically significant increment in total cell numbers compared with NonTg controls. The increase was at the expense of both CD8^+^ and CD4^+^ total T cells (Figure 2B and 2C), but not of other lymphocyte subsets such as NK, NKT or B cells (Supplementary Figure 1). Regarding lymphocyte cell subset percentages, no significant differences were observed between shCD5EμTg and NonTg controls; the only exception being CD4^+^ T cells, which were increased in TdLN from shCD5EμTg mice (Figure 2C right). Worth noting was the fact that, although the percentage and number of total CD4^+^ T cells was increased in the TdLN of shCD5EμTg mice, the percentage of CD4^+^ cells with regulatory phenotype (CD25^+^FoxP3^+^) was found to be reduced in both TdLN and cLN compared to NonTg controls (Figure 2D). However, the suppressive activity of T_reg_ did not differ between shCD5EμTg and NonTg controls (Supplementary Figure 2). Overall, the quantitative changes observed in CD4^+^, CD8^+^, and T_reg_ T-cell subset composition of TdLN from shCD5EμTg mice would be compatible with a more efficient anti-tumor response. It is worth mentioning on this regard, that similar TdLN changes were observed when shCD5EμTg mice were challenged with tumor cells for which no efficient antitumor response was observed (namely, MCA-205) (Supplementary Figure 3). This would indicate that tumor-related factors may be behind the inefficient anti-tumor response mounted by shCD5EμTg mice against certain tumor cell types.

### shCD5EμTg mice exhibit enhanced tumor specific and unspecific cytotoxic responses

**Figure 3:**
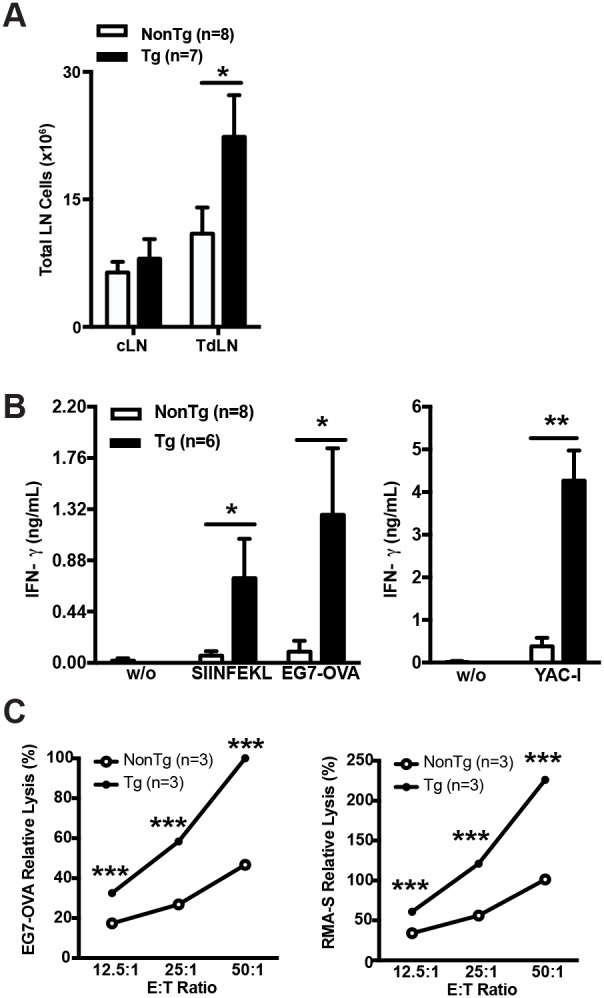
shCD5EμTg mice exhibit enhanced innate and adaptive anti-tumor responses **(A)** Total cell numbers from cLN and TdLN of shCD5EμTg (n = 7) and NonTg (n = 8) mice challenged with 5×10^4^ EG7-OVA cells. **(B)** TdLN cells from the same mice as in A were co-cultured in the absence (w/o) or presence of SIINFEKL peptide, and irradiated EG7-OVA cells **(left panel)** or YAC-1 cells **(right panel)** for 48 h. IFN-γ levels (ng/ml) in culture supernatants are represented. Data are presented as mean ± SEM. **(C)** Relative lysis of EG7-OVA **(left panel)** and RMA-S **(right panel)** by LN cells from EG7-OVA tumor bearing mice. 5×10^4^ irradiated tumor cells were co-cultured for 5 h at the indicated effector:target (E:T) ratios with pooled LN cells from shCD5EμTg (n = 3) and NonTg (n = 3) mice challenged with EG7-OVA cells for 15 days. Data are presented as mean ± SEM from triplicates. ^*^p<0.05; ^**^p<0.01; ^***^p<0.0001 (unpaired *t* test).

To analyze the specificity of their anti-tumor immune responses, shCD5EμTg and NonTg control mice were again challenged with EG7-OVA cells for further analyses of TdLN and cLN cell suspensions at the end of the follow-up period. As previously shown for B16-F0 cells, a statistically significant increase of total cells was observed in TdLN from EG7-OVA-challenged shCD5EμTg mice (Figure 3A). The same LN cell suspensions were further cultured *in vitro* in the presence or absence of OVA-specific MHC class I-restricted SIINFEKL peptide or irradiated EG7-OVA cells. At 48 h post-stimulation, IFN-γ production was assessed in culture supernatants by ELISA. As shown by Figure 3B, left panel, statistically significant higher levels of IFN-γ were detected for TdLN cells from shCD5EμTg mice under all the stimulatory conditions tested, compared with those of NonTg mice. The analysis of cLN cells from both mouse groups did not result in detectable IFN-γ levels following stimulation (data not shown). Interestingly, similar IFN-γ results were obtained when the same cLN and TdLN cell suspensions were challenged with irradiated allogeneic YAC-1 cells, a mouse thymoma of A/Sn origin (H2a) commonly used for assaying NK-mediated cytotoxicity (Figure 3B, right panel). This would indicate that tumor-challenged shCD5EμTg mice exhibit increased both specific and non-specific anti-tumor responses. Further evidence on the latter regard was obtained by performing lytic assays with spleen cell suspensions from EG7-OVA-bearing shCD5EμTg and NonTg mice. As illustrated by Figure 3C, shCD5EμTg mice bearing EG7-OVA tumors displayed significantly higher lytic activity not only against EG7-OVA cells (Figure 3C, left panel) but also RMA-S cells (Figure 3C, right panel).

### Exogenous administration of rshCD5 to WT mice induces similar TdLN changes to those observed in shCD5EμTg mice

**Figure 4:**
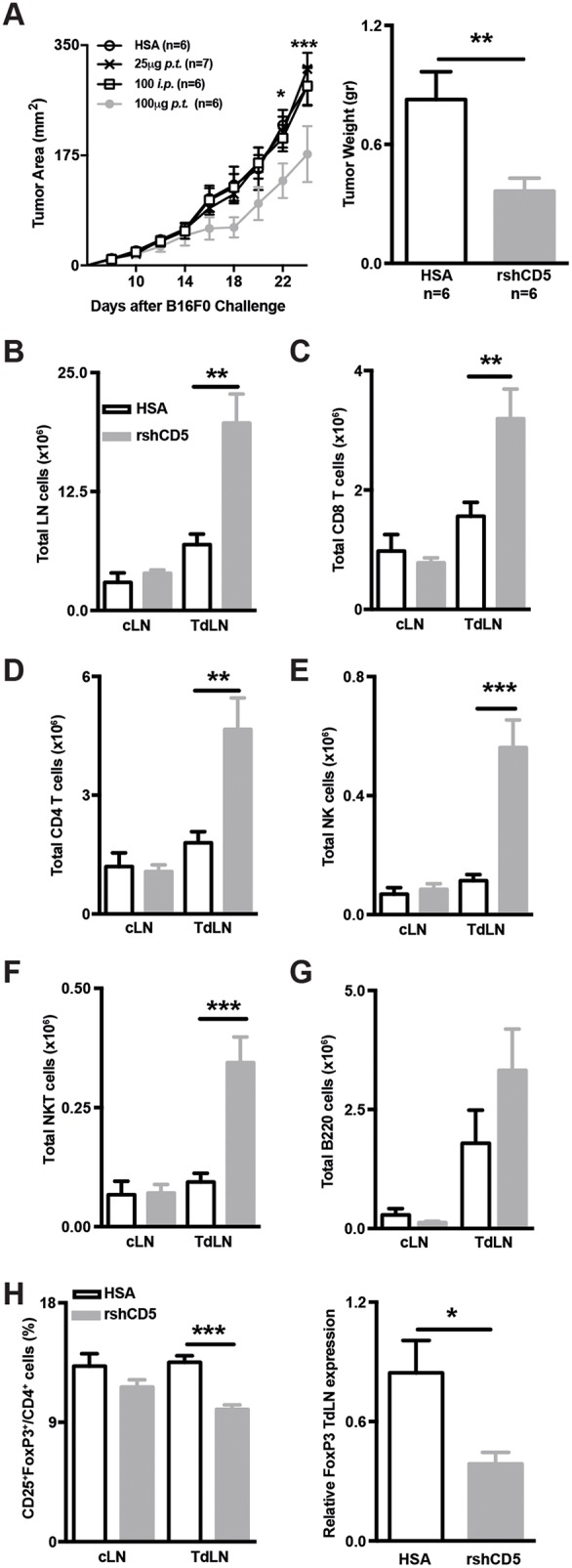
Exogenous administration of rshCD5 to WT mice mimics the anti-tumor effects of shCD5EμTg mice **(A)** Tumor growth curve in HSA- and rshCD5-treated WT C57BL/6J mice (n = 6-7/group) injected *s.c.* with 5×10^4^ B16-F0 cells **(left panel)**. HSA (100 μg *p.t.*) or rshCD5 (25 or 100 μg, *p.t.* or *i.p*.) were administered every 48 h starting when tumor size was approx. 9-12 mm^2^ (~day 7 post-injection). Tumor weight at day 23 is also represented **(right panel)**. **(B)** Total cell numbers in cLN and TdLN from the same mice as in (A). **(C-G)** Total number of CD8^+^ (C), CD4^+^ (D) cells, NK (E), NKT (F) and B220^+^ (G) cells in cLN and TdLN from the same mice as in (A). **(H)** Percentage of CD25^+^FoxP3^+^ cells in CD4^+^ from cLN and TdLN from the same mice as in A **(left panel)**. The relative FoxP3 mRNA expression in TdLN cells from the same mice is represented **(right)**. Data are presented as mean ± SEM. ^*^p< 0.05, ^**^p< 0.01, and ^***^p<0.0001 (unpaired *t* test).

In order to exclude transgenesis artifacts and to get closer to a clinical application, the effects of rshCD5 infusion to WT mice bearing B16-F0 tumors was further explored. To this end, rshCD5 or human serum albumin (HSA) were administered every other day by two different routes (*i.p.* or *p.t.*) at two different doses (100 μg and 25 μg) starting when tumors were ~9-12 mm^2^ in size (~day 7-8). As illustrated by Figure 4A, peritumoral *(p.t.*) administration of high rshCD5 doses (100 μg/mice) induced a statistically significant slower tumor growth and lower tumor weight compared with those from HSA-treated mice. On the contrary, no significant effects on tumor growth were observed when rshCD5 was administered *p.t.* at low doses (25 μg) or *i.p.* even at high doses (100 μg) (Figure 4A). Significantly higher total cell numbers were observed in TdLN but not cLN cell suspensions from mice *p.t.* treated with rshCD5 (100 μg) compared with HSA-treated ones (Figure 4B). The increment in total cell numbers from TdLN was at the expense of total CD8^+^ and CD4^+^ T cells as well as NK and NKT cell numbers (Figure 4C–4F). No statistically significant differences were observed regarding total B-cell numbers (Figure 4G). The percentage of CD25^+^FoxP3^+^ cells within the CD4^+^ subset was significantly reduced in TdLN but not cLN from rshCD5-treated mice compared with HSA-treated ones (Figure 4H). This finding was validated by the analysis of FoxP3 mRNA levels in TdLN from *p.t.* rshCD5-treated versus HSA-treated mice (Figure 4H). Taken together, these results indicate that *p.t.* infusion of rshCD5 reproduces most of the observations made in shCD5EμTg regarding slower tumor growth and cellularity changes in TdLN.

### Recombinant shCD5 impacts in T_reg_ and T_H_1 *in vitro* polarization of naïve T cells

**Figure 5:**
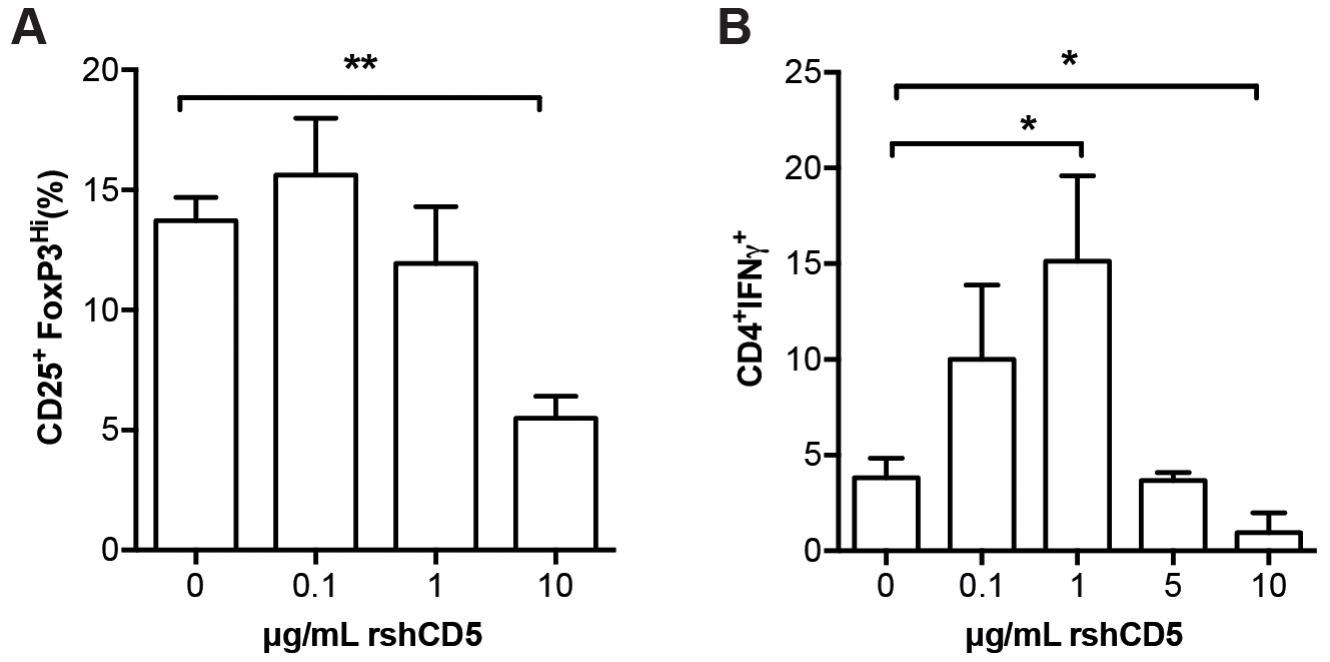
Effect of rshCD5 on *in vitro* T_reg_ and T_H_1 polarization Sorted naïve T cells CD4^+^CD25^-^CD62L^hi^CD44^lo^ cells (1 × 10^5^) from C57BL/6 mice were activated for 96 h in triplicate with plate-bound α-CD3 (2 μg/mL) and soluble α-CD28 mAb (0.5 μg/mL) under **(A)** T_reg_ polarization (α-IL-4 mAb, 1 μg/mL; TGF-β, 2 ng/mL; IL-2, 5 ng/mL; and of α-IFN-γ, 1 μg/mL) or **(B)** T_H_1 polarization (α-IL-4 mAb, 10 μg/mL; IL-2, 5 ng/mL; and IL-12, 10 ng/mL) conditions in the presence of different amounts of rshCD5 (0-10 μg/mL). Then cells were (A) stained for surface CD4, CD25 and intracellular FoxP3 expression or (B) re-stimulated for 5 h with PMA (80 nM) and Ionomycin (1 μg/mL) in the presence of 2 μM Monensin followed by surface CD4 and intracellular IFN-γ staining, for further flow cytometry analyses. Data represent the mean percentage of double-positive cells (mean ± SD) from three experiments (A) or one representative experiment of two (B) performed. ^*^, p<0.05; ^**^, p<0.01 (Unpaired *t*-test).

At this point, we decided to evaluate if shCD5 could interfere with the normal T_reg_ cell induction. To do so *in vitro* polarization assays were performed with WT naïve T-cells. Under T_reg_ polarization conditions, rshCD5 induced a dose-dependent decrease in the percentage of CD25^+^FoxP3^hi^ cells (Figure 5A). This result is in agreement with the lower percentage of T_reg_ cells observed both in the shCD5EμTg and rshCD5-treated mice. On the other hand, when the same cells were polarized towards a T_H_1 phenotype, a trend towards increased proportion of CD4^+^IFNγ^+^ cells was observed in the presence of relative low rshCD5 concentrations (0.1-1 μg/mL), which reached statistical significance at 1 μg/mL. Inconsistently, at higher rshCD5 concentrations (5 and 10 μg/mL) no such enhancement effects were observed (Figure 5B). Although the bi-phasic effect of rshCD5 on T_H_1 polarization *in vitro* will require further elucidation, the more efficient T_H_1 polarization observed at low rshCD5 concentration (which are closer to the ones achieved *in vivo*) are also compatible with the enhanced anti-tumor response observed both in shCD5EμTg and rshCD5-treated mice.

### NK cells are critical for the shCD5-induced anti-tumor effects

**Figure 6:**
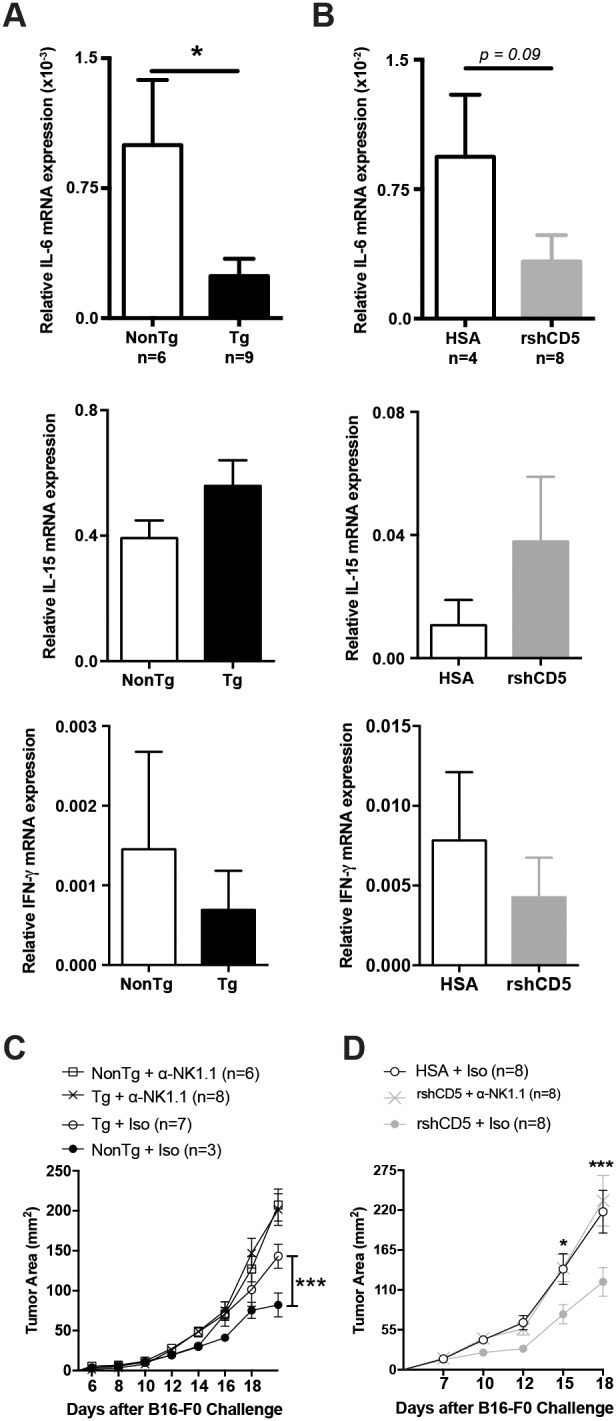
Abrogation of shCD5-mediated anti-tumor effects by NK cell depletion **(A)** Relative IL-6 **(top)**, IL-15 **(middle)** and IFN-γ **(bottom)** mRNA expression in tumors from shCD5EμTg (n = 9) and NonTg mice (n = 6) challenged with B16-F0 cells as in Figure 1A. Data are presented as mean ± SEM. ^*^p< 0.05 (unpaired *t* test). **(B)** Relative IL-6 **(top)**, IL-15 **(middle)** and IFN-γ **(bottom)** mRNA expression in tumors from rshCD5- (n = 8) or HSA- (n = 4) treated WT mice challenged with B16-F0 cells as in Figure 4A. Data are presented as mean ± SEM. **(C)** Tumor growth curves of shCD5EμTg and NonTg mice treated with anti-NK1.1-PK136 or IgG2a isotype control antibodies before and after challenge with 5×10^4^ B16-F0 cells. **(D)** Tumor growth curves of B16-F0-challenged (5×10^4^ cells) C57BL/6J mice treated with anti-NK1.1-PK136 or IgG2a isotype before and after starting administration of rshCD5 or HSA (100 μg *p.t.*) when time tumor diameter was ~9-12 mm^2^ (~day 7 post-injection). Data are presented as mean ± SEM. ^*^p< 0.05, ^***^p<0.0001 (two-way ANOVA statistical test).

The analysis of intratumor cytokine mRNA expression levels in B16-F0-challenged shCD5EμTg and NonTg mice showed a trend to reduced IL-6 but increased IL-15 mRNA levels, which only reached statistical significance in the former case. No significant differences were observed for other relevant cytokines (IFN-γ, IL-10, and IL-22) (Figure 6A, and data not shown). A similar result was obtained when rshCD5 was administrated exogenously (*p.t.*) to WT mice (Figure 6B). The fact that IL-6 and IL-15 are inhibitor and activator, respectively, of NK effector functions [25, 26], together with the enhanced IFN-γ release by TdLN cells from tumor-bearing shCD5EμTg mice under both specific and non-specific re-stimulation conditions, prompted us to confirm a putative implication of NK cells in the anti-tumor effects induced by transgenic or exogenous shCD5. To this end, shCD5EμTg and Non-Tg mice were treated *i.p.* with an NK cell-depleting mAb (anti-NK1.1, clone PK136) or an isotype control every other day, starting with two consecutive doses two days before B16-F0 cells implantation. As illustrated by Figure 6C, NK cell-depletion abrogated the statistically significant differences in tumor growth observed between shCD5EμTg and NonTg mice treated with the isotype control antibody. Consistently, similar NK cell-depletion treatment also abrogated the enhanced anti-tumor effect of *p.t.* rshCD5 administration to tumor-bearing WT mice (Figure 6D), thus confirming the relevant role played by NK cells in shCD5-mediated outcome.

## DISCUSSION

The present work further supports available evidence on the involvement of CD5 –a negative modulator of T-cell activation- in the fine-tuning of immune responses in general and of anti-tumor responses in particular [20, 27–29]. Our study shows that increasing the circulating/local levels of a soluble form of human CD5 (shCD5) might result in both specific and non-specific enhancement of immune responses to cancer cells. This was achieved by using two different experimental approaches, one involving the use of a homozygous transgenic mouse line (shCD5EμTg) expressing circulating shCD5 [24], and the other involving repeated local infusions of purified recombinant shCD5 protein (rshCD5). Thanks to the inter-species recognition of the receptor-ligand interactions between mouse and human CD5 [24], functional blockade of those interactions (“decoy receptor effect”) is expected from both experimental approaches. Indeed, mouse and human CD5 are highly homologous receptors, with a gradient of amino acid homology from 60.2% for the N-terminal domain D1, 66.6% for domain D2, 74.2% for domain D3 and 92.7% for the transmembrane and the C-terminal cytoplasmic region. Thus, the most conserved extracellular domain (D3) would be the most suited for interaction with putative CD5 ligand/s conserved interspecies. While minor amino acid sequence variation could deeply alter receptor function, it also true that amino acid positions important for protein structure and/or function are relatively well conserved across species. This is well exemplified by the interaction of the closely related receptor CD6 with its ligand (CD166/ALCAM). The most membrane-proximal extracellular domain of human CD6 (D3) interacts with the most amino-terminal domain (D1) of human and mouse CD166/ALCAM and this is achieved through conserved key amino acids at the interaction interface [30].

Previous characterization of heterozygous shCD5EμTg mice showed that they have significantly reduced proportions of spleen and lymph node T_reg_ cells (CD4^+^CD25^+^FoxP3^+^), and of peritoneal IL-10-producing CD5^+^ B (B10) cells, as well as increased proportions of spleen NKT cells [24]. Similar phenotypical changes were observed in WT mice following repeated *i.p*. administration of rshCD5 protein [24]. The functional relevance of these phenotypic changes was evidenced by delayed growth of B16-F0 melanoma tumors in shCD5EμTg mice [24]. By using homozygous shCD5EμTg mice, we show here that the enhanced anti-melanoma response is not melanoma-specific since it also extends to cancer cells of lymphoid origin (namely, EG7 thymoma). However, no delayed tumor growth was observed for other cancer cells of different lineages (RMA-S lymphoma, MCA-205 sarcoma, and MC-38 colon carcinoma), thus indicating that differences in tumor antigenicity or other tumor-related factors should also be taken into consideration.

In an attempt to unravel the basis by which shCD5EμTg mice displayed slower B16-F0 or EG7-OVA tumor growth, we found that their TdLNs had significant higher total cell numbers compared to NonTg controls. This included higher total numbers of CD4^+^ and CD8^+^ T cells with lower percentage of T_reg_ cells. The *ex vivo* re-stimulation of TdLN cells from EG7-OVA tumor-bearing mice with SIINFEKL -a specific CD8^+^ T cell ovalbumin-derived peptide- showed that shCD5EμTg mice were more responsive than NonTg controls, as deduced from increased IFN-γ release. Interestingly, even higher IFN-γ levels were observed when the same TdLN cells were re-stimulated with EG7-OVA cells or unrelated YAC-1 tumor cells. Concomitantly, stimulation of cLN cells rendered undetectable IFN-γ levels. These results advocate for the existence of not only increased OVA-specific CD8^+^-mediated responses in shCD5EμTg mice, but increased unspecific anti-tumor responses as well. The fact that IFN-γ levels following SIINFEKL stimulation were the lowest of all the stimuli tried allow speculating that CD8^+^ cells could represent only a minor source of IFN-γ. By contrast, the highest levels of IFN-γ observed following stimulation with YAC-1 cells support a greater contribution of non-specific players such as NK cells. This indicates that the non-specific anti-tumor response is more relevant than the specific anti-tumor response in shCD5EμTg mice after the tumor challenge.

The demonstration that the delayed tumor growth and the changes in TdLN composition observed in shCD5EμTg mice were not transgenesis artifacts but the effect of shCD5 expression came from infusing WT mice with rshCD5 protein. Local (*p.t.*) treatment proved to be the most effective route, lowering tumor growth rate and weight in a dose-dependent manner. The presence of rshCD5 in the tumor environment was able to mimic the changes in TdLNs observed in shCD5EμTg mice (increased total CD4^+^ and CD8^+^ T-cell numbers and decreased proportion of T_reg_ cells), but also to induce increased total NK and NKT cells –two cell types involved in non-specific anti-tumor responses. This scenario of increased specific and non-specific effector cells (CD8^+^T, NK and NKT) and lowered regulatory cells (T_reg_) would suit the observed anti-tumor effects. However, it should be taken into consideration that tumor-induced T_reg_ cells are known to differentially affect NK cells activated in the presence or absence of tumor cells [31].

An alternative or complementary scenario would relate to the detection of lower intratumor IL-6 mRNA levels in tumors from both shCD5EμTg mice and rshCD5-treated WT mice. IL-6 is a pro-inflammatory cytokine normally released by several cell types (e.g., monocytes, T cells, fibroblasts, epithelial and endothelial cells), and whose aberrant expression is associated with the growth, metastasis, and chemotherapeutic resistance in a wide range of cancers [32]. Inhibition of NK cell cytotoxicity by IL-6 has been reported in both mice and humans [25]. So, increased NK activity could be behind the observed exacerbated anti-tumor responses in transgenic and rshCD5-treated mice. This was supported by the fact that NK cell depletion *in vivo* fully reversed the beneficial effects of both transgenic and *p.t.* infused shCD5. This by no means totally excludes a putative contribution of other effector cells (namely CD8^+^ T cells).

Intriguingly, a recent report claims CD5 as a novel ligand for IL-6 [33]. The authors show that IL-6 activates STAT3 in CD5^+^ B cells in an IL-6 receptor-independent manner. This in turn promotes IL-10 expression and self-expansion of regulatory CD5^+^CD19^+^ B cells (B10) in tumor microenvironment, resulting in promotion of tumor growth [33]. Based on this finding, it could be hypothesized that IL-6 sequestration by shCD5 would result in limiting the number and/or function of tumor-associated B10 cells. In this scenario, a blockade of IL-6-IL-6 receptor (IL-6R) (membrane-bound or soluble) interaction [34] by the presence of shCD5 would limit any IL-6 activation positive feedback loop [33] resulting in low IL-6 mRNA levels.

In conclusion, the data obtained from melanoma and thymoma tumor models support the notion that local/systemic shCD5 would favor accumulation of innate and adaptive immune effector cells (by increased cell proliferation and/or cell recruitment) into TdLN, while limiting that of cells with regulatory function. This would be likely achieved by interfering (decoy receptor effect) with the interactions between CD5 and still ill-defined membrane-bound and/or soluble ligand/s. There is, however, also the possibility that shCD5 effects could be mediated by decoying not only CD5 signaling but also that of other activator/suppressor cell surface receptors. Nevertheless, previous reports on CD5-deficient mice provide evidence that sole abrogation of CD5 signaling leads to enhanced anti-tumor response [17]. Our results with transgenic or exogenously infused rshCD5 are in full agreement with such evidence, and make unlikely the involvement of other receptors in the anti-tumor effects observed. Whatever the case, the results warrant future studies exploring CD5 targeting to improve the efficacy of currently available immunotherapeutic approaches against cancer such as IL-10 [35], TGF-β inhibitors [36] or IL-2/anti-IL-2 mAb immunocomplexes [37].

## MATERIALS AND METHODS

### Tumor cell lines

Melanoma B16-F0 cells were kindly provided by Dr. Ramón Alemany (Institut Català d’Oncologia, L’Hospitalet de Llobregat, Spain). RMA-S lymphoma cells were a kind gift from Dr. Pilar Lauzurica (Instituto de Salud Carlos III, Madrid, Spain). Thymoma EG7-OVA cells were kindly provided by Dr. Elio Schouppe (Vrije Universiteit Brussel, Brussels, Belgium). Colon adenocarcinoma MC-38 cells and NK-sensitive YAC-1 cells were kindly provided by Dr. Pedro Berraondo and Dr. Pablo Sarobe (Center for Applied Medical Research, Pamplona, Spain). B16-F0 cells were grown in DMEM/F12 (Gibco Life Science) supplemented with antibiotics (100 U/mL penicillin, 100 μg/mL streptomycin) and 10% heat-inactivated FBS (Walkersville). EG7-OVA, RMA-S, MCA-205, MC-38 and YAC-1 cells were grown in RPMI 1640 supplemented with antibiotics (100 U/mL penicillin, 100 μg/mL streptomycin), 2 mM L-glutamine, 10 mM HEPES, 0.05 mM β-mercaptoethanol and 10% heat-inactivated FBS.

### Mice

*In vivo* studies were carried out at the animal facilities of the School of Medicine of the University of Barcelona under protocols approved by the Ethics Committee for Animal Research of the University of Barcelona (permits number 740/14, 741/14 and 54/16). Homozygous shCD5EμTg transgenic mice of C57BL/6 genetic background were obtained by intercrossing of previously reported heterozygous mice [24]. The homozygosity of shCD5EμTg mice was indirectly ascertained from their offspring breeding homozygous candidates with non-transgenic mice (NonTg). NonTg mice used for comparative purposes came from the same common heterozygous ancestors as the shCD5EμTg mice and were kept under the same housing conditions as the latter. For some experiments WT C57BL/6J mice were purchased from Charles River. All animals were maintained under conventional (non-specific-pathogen-free) housing conditions.

### Recombinant proteins

Production of purified rshCD5 protein (PBS with 10% glycerol, pH 7.4) was carried out as previously reported [38] but using stable transfected SURE CHO-M Cell line™ clones from the Selexis SUREtechnology Platform™ (Geneva, Switzerland) and subjecting their serum-free supernatants to size-exclusion chromatography protocols developed at PX´Therapeutics (Grenoble, France). Recombinant Human Serum Albumin (HSA; in PBS with 10% glycerol, pH 7.4) was from Sigma-Aldrich.

### Flow cytometry analysis

The following mAbs were used to characterize mouse lymphocyte subpopulations: Fluorescein isothiocyanate (FITC)-labeled anti-CD4 (RM4-5, Tonbo Bioscience); Phycoerythrin (PE)-labeled anti-NK1.1 (PK136, BD Biosciences), anti-FoxP3 (FJK-16s), and anti-B220 (RA3-6B2, Tonbo Bioscience); PerCP-Cy5.5-labeled anti-CD3 (145-2C11, Tonbo Bioscience); Allophycocyanin (APC)-labeled anti-CD8 (53-6.7, Tonbo Bioscience) and anti-CD25 (PC61, BD Pharmingen); violetFluor 450-labeled anti-CD8 (53-6.7, Tonbo Bioscience). Before surface staining with predetermined optimal concentrations of each mAb, cell samples (1×10^6^) were blocked by incubation with FBS 10% in PBS and anti-mouse CD16/CD32 (Fc Shield, 2.4G2, Tonbo Bioscience) for 30 min at 4° C. For intracellular FoxP3 staining the PE-labeled anti-mouse/rat Treg Staining Kit (eBioscience) was used according to the manufacturer's instructions. Nine-color flow cytometry was performed on a BD FACSCanto II flow cytometer (Becton Dickinson, US) and data analyzed using FlowJo software (Tree Star, USA). Each analysis shown represents ≥100,000 events within the live lymphocyte gate.

### T_reg_ suppression assay

CD4^+^CD25^−^ (T_conv_) and CD4^+^CD25^+^ (T_reg_) T cells were magnetically separated from LN specimens by autoMACS Pro Separator using the mouse CD4^+^CD25^+^ Regulatory T Cell Isolation Kit (Milteny Biotec). Upon CFSE-labeling, cells were co-cultured for 72 h at 2:1 T_con_:T_reg_ ratio (1×10^5^:5×10^4^) in 96-well U-bottom plates pre-coated with 10 μg/mL anti-CD3 mAb (145.2C11; Tonbo Bioscience) or isotype control (Armenian hamster IgG isotype, BioLegend) plus 1 μg/mL soluble anti-CD28 (37.51; Tonbo Bioscience). T_conv_ cell proliferation was analysed by determining the percentage of CFSE^low^ cells in a BD FACSCanto II flow cytometer.

### *In vivo* tumor growth assays

Mice were challenged by *s.c.* injection of B16-F0, EG7-OVA, RMA-S, MCA-205 or MC-38 cells (5×10^4^) on the right flank with a 23-gauge needle. Tumors were measured every other day with a Vernier caliper, and the area (length by width of the tumors, mm^2^) averaged. For therapeutic assays, rshCD5 or HSA (25 μg or 100 μg each per mouse) was administrated *i.p.* or *p.t.* every 48h, starting when tumors were ~9-12 mm^2^ in size. In NK cell depletion experiments with shCD5EμTg and NonTg mice, animals were *i.p.* administered with 200 μg of NK1.1-PK136 or rat IgG_2a_ isotype control (BioXCell) two days prior to tumor cell implantation. The following two days 100 μg doses of the same antibodies were repeated. After this, 100 μg doses were administered every 48 h until the end of the experiment. In the case of rshCD5- or HSA-treated WT mice, animals received 200 μg *i.p.* of NK1.1-PK136 or IgG_2a_ isotype control when tumors reached approximately ~9-12 mm^2^. The following two days, a 100 μg dose of the same antibodies was repeated. After this, 100 μg doses were administered every 48 h until the end of the experiment, concurrently with rshCD5 or HSA protein administration.

### Cytotoxicity assays

γ-irradiated (1 cycle of 2,000 rads) tumor cells were seeded at 5×10^4^ cells/well in U-bottomed 96-well plates in RPMI 1640 supplemented with antibiotics but no FCS. Splenocytes obtained from tumor-bearing mice following tissue disaggregation through a cell strainer (Biologix group Ltd) and further red blood cell lysis (Red Blood cell lysis buffer; eBioscience) were then added at different effector:target (E:T) ratios and incubated for 5 h. Tumor cell lysis was measured using the CytoTox-ONE™ Homogeneous Membrane Integrity Assay (Promega) according to manufacturer's instructions and a microplate luminometry reader (Bio-TEK). Percentage of relative lysis was calculated as follows: % specific cytotoxicity = [experimental lysis - spontaneous lysis]/[maximal lysis - spontaneous lysis] × 100.

### IFN-γ measurement

IFN-γ levels in the cell culture supernatants were determined by BD OptEIA™ - Mouse ELISA Set (BD Biosciences) following manufacturer's instructions. To this end, TdLN or cLN cells (2×10^5^) were co-cultured for 48 h with EG7-OVA or YAC-1 irradiated cells (2×10^4^) or OVA-specific SIINFEKL (5 μg, Sigma) peptide at 37° C and 5% CO_2_.

### Cytokine mRNA levels measurement

Total RNA from tumor and LN samples was isolated by a TRIzol (Invitrogen)/chloroform (AnalaR NORMAPUR) procedure, and stored at -80° C until use. Further purification of RNA was performed with the PureLink RNA Mini Kit (Ambion, Life technologies) according to manufacturer's instructions, and RNA purity assessed by the 260/280nm ratio, with samples being studied only when ratio was between 1.8 and 2.2. Total cDNA was synthesized using the High capacity cDNA Reverse Transcription kit (Thermofisher) according to manufacturer's instructions. Samples were kept at 4° C (or -20° C) until quantitative real-time PCR (qPCR) performed. To this end, IL-6 (Mm01210733_m1), IL-10 (Mm01288386_m1), FoxP3 (Mm00475162_m1), IL-15 (Mm00434210_m1) and IL-22 (Mm01226722_g1) specific Taqman probes and Taqman Fast universal PCR master Mix (Life Technologies/ThermoFisher) were used. Gene expression was determined by using Ct values inferior or equal to 30 cycles. The results were normalized with the expression values of the non-inducible gene Gliceraldehide-3-phosphate dehydrogenase (GADPH, Mm99999915_g1, Life Technologies/ThermoFisher), using the 2^ΔCt^ formula, where ΔCt=Ct (GADPH)-Ct (gene of interest). Results are represented as relative values.

### *In vitro* T_reg_ and T_H_1 polarization of naïve T cells

FACS sorted (FACSAria) naïve T CD4^+^CD25^-^CD62L^hi^CD44^lo^ cells (1 × 10^5^) from C57BL/6 mice were activated for 96 h in triplicate in 96-well U-bottom plates pre-coated α-CD3 (2 μg/mL) and soluble α-CD28 mAb (0.5 μg/mL) under T_reg_ polarization (α-IL-4 mAb, 1 μg/mL; TGF-β, 2 ng/mL; IL-2, 5 ng/mL; and of α-IFN-γ, 1 μg/mL) or T_H_1 polarization (α-IL-4 mAb, 10 μg/mL; IL-2, 5 ng/mL; and IL-12, 10 ng/mL) conditions in the presence of different amounts of rshCD5 (0-10 μg/mL). Cells were stained for surface CD4 and intracellular FoxP3 expression for T_reg_ analysis. For T_H_1 analysis, cells were re-stimulated for 5 h with PMA (80 nM) and Ionomycin (1 μg/mL) in the presence of 2 μM Monensin followed by surface CD4 and intracellular IFN-γ staining, for further analyses with a BD FACSCanto II flow cytometer.

### Statistical analyses

Statistical significance of differences between groups was determined using Student's *t* test or ANOVA test, unless stated otherwise, using GraphPad Prism 5.03 software. In all experiments, differences were considered statistically significant when *p*<0.05.

## SUPPLEMENTARY MATERIALS FIGURES



## Abbreviations

rshCD5: recombinant soluble human CD5
nonTg: non-transgenic mice
cLN: contra-lateral lymph node
TdLN: tumor draining lymph node
mAb: monoclonal antibody
WT: wild type mice, *p.t*, subcutaneous peritumoral
*i.p.*: intraperitoneal
T_reg_: T regulatory cells
NK: Natural killer cells
NKT: Natural killer T cells
TCR: T-cell receptor
BCR: B-cell receptor
