# Supplementary material for: Immunomodulatory effects of soluble CD5 on experimental tumor models
Source: Oncotarget. 2017 Nov 20;8(64):108156–69. doi: 10.18632/oncotarget.22564 (PMC5746133; doi:10.18632/oncotarget.22564)
Supplement: Supplementary file 1 [file oncotarget-08-108156-s001.pdf]

Immunomodulatory effects of soluble CD5 on experimental tumor models

SUPPLEMENTARY MATERIALS

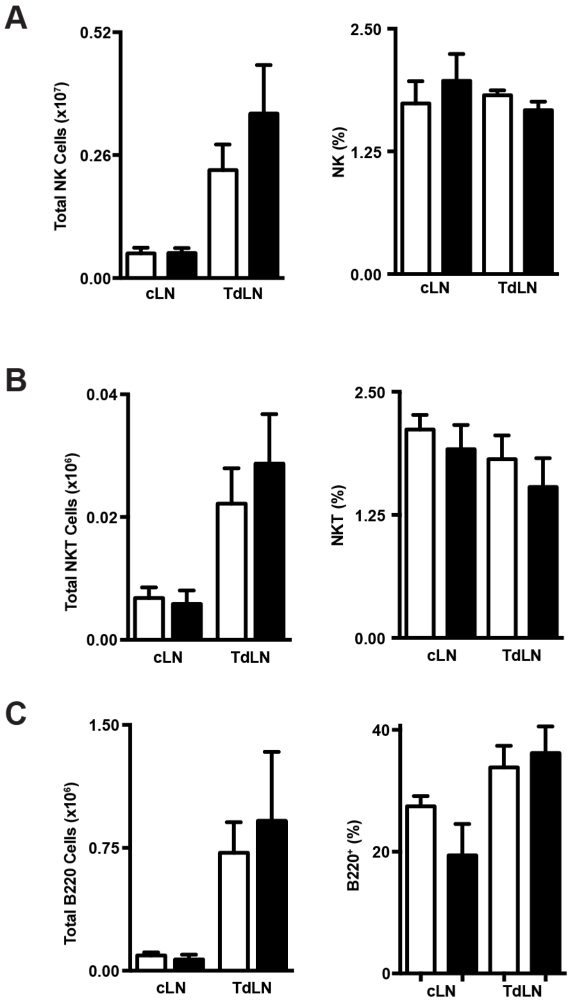

Supplementary Figure 1: Absence of significant LN changes in NK, NKT and B cell populations of tumor-challenged shCD5EμTg mice. Total cell numbers **(left)** and percentage **(right)** of NK **(A)**, NKT **(B)** and B **(C)** cells from TdLN and cLN from the same B16-F0-challenged shCD5EμTg mice as in Figure 2. Values are represented as mean ± SEM.

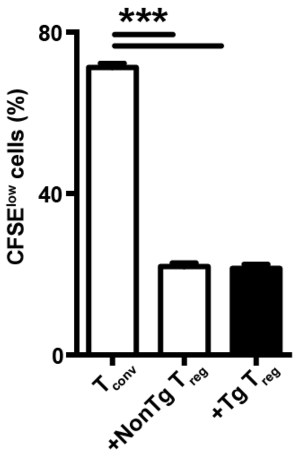

Supplementary Figure 2: Absence of significant differences in the suppressive activity of T_{reg} cells from shCD5EμTg and NonTg mice. CFSE-stained T_{conv} cells (1x10^{5}cells) from LN of NonTg mice were cultured in anti-CD3 mAb–coated plates plus soluble anti-CD28 mAb (1 μg/ml) in the absence or presence of T_{reg} cells from NonTg and shCD5EμTg mice LN at 2:1 ratio (5x10^{4}cells). Shown is the percentage of CFSE^{low} lymphocytes at day 3 of culture from one representative experiment out of three performed. Values are represented as mean ± SEM of duplicates from cells pooled from 3 mice of each phenotype. ***, p <0.0001 (unpaired *t* test).

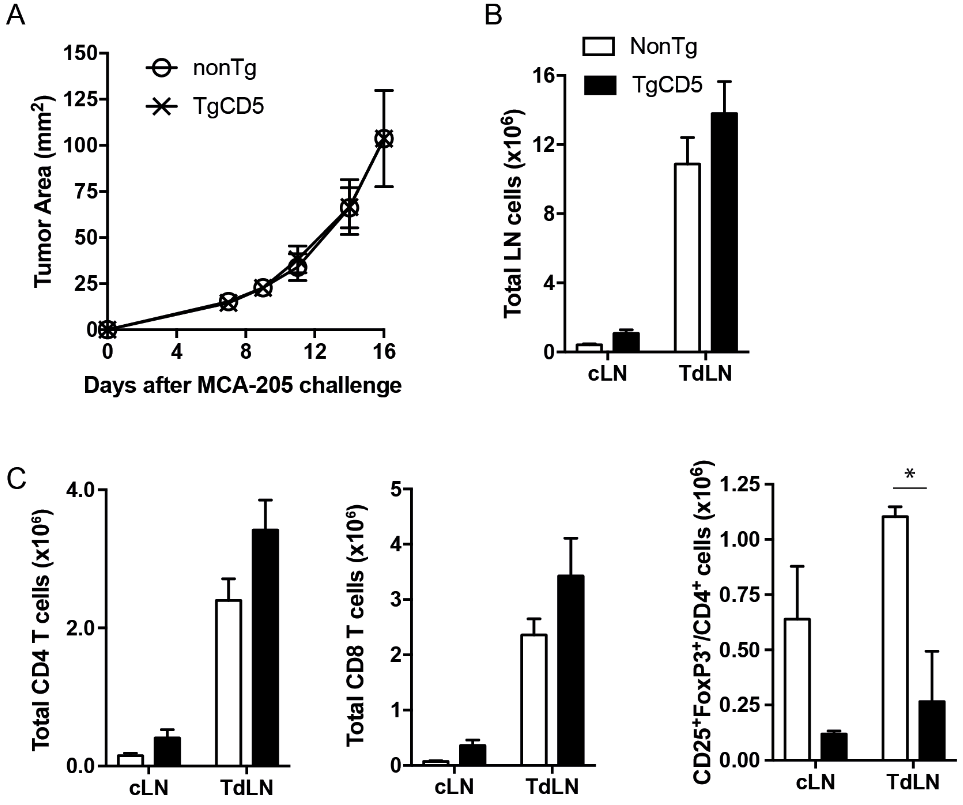

Supplementary Figure 3: Analysis of lymphocyte changes in cLN and TdLN from MCA-205-challenged shCD5EμTg mice. **(A)** shCD5EμTg (n = 4) and NonTg (n = 4) mice were injected *s.c.* with MCA-205 cells (5 x 10^4) and tumor area measured every other day. **(B-C)** Total cell numbers, and total CD4$^+$, CD8$^+$ and T_{reg} (CD25$^+$FoxP3$^+$CD4$^+$) T cells from cLN and TdLN of the same mice as in (A) were counted from single cell suspensions and analyzed by flow cytometry at day 16. Values are represented as mean ± SEM. *p< 0.05 (unpaired *t* test).
